# Short-Term Trends in Economic Burden and Catastrophic Costs of Type 2 Diabetes Mellitus in Rural Southwest China

**DOI:** 10.1155/2019/9626413

**Published:** 2019-08-01

**Authors:** Hui-fang Li, Le Cai, Allison Rabkin Golden

**Affiliations:** ^1^School of Public Health, Kunming Medical University, 1168 Yu Hua Street Chun Rong Road, Cheng Gong New City, Kunming 650500, China; ^2^The First Affiliated Hospital of Kunming Medical University, Renmin Western Road, Kunming, China

## Abstract

**Objectives:**

This study is aimed at gaining insights on the changing prevalence, economic burden, and catastrophic costs of diabetes in rural southwest China.

**Materials and Methods:**

Data were collected from two cross-sectional health interviews and examination surveys among individuals aged ≥ 35 years in rural Yunnan Province. A prevalence-based cost-of-illness method was used to estimate the cost of diabetes. Information about the participants' demographic characteristics and economic consequences of diabetes was obtained using a standard questionnaire. Fasting blood sugar levels were recorded for each study participant.

**Results:**

During the study period, the overall prevalence of diabetes increased from 7.7% to 9.5% (*P* < 0.01) and the economic cost of diabetes increased 1.52-fold. The largest increases were observed in hospital costs (1.77-fold increase), while unit medication costs fell by 18.6%. Both in 2009 and in 2016, males had higher overall direct and indirect costs of diabetes than females (*P* < 0.05). Direct costs represented the largest component of economic cost of diabetes while hospital costs were the main drivers of direct medical expenditures, accounting for 66.2% of the total direct costs in 2009 and 75.9% in 2016. The incidence of household catastrophic health payment and household impoverishment due to diabetes was 24.0% and 17.9% in 2009 and 23.6% and 17.6% in 2016, respectively. These rates did not differ between the two survey years (*P* > 0.05).

**Conclusions:**

The prevalence and economic burden of diabetes increased substantially from 2009 to 2016 in rural southwest China. The findings underscore an urgent need for the government to invest more financial resources in the prevention of diabetes and improvement of access to affordable medication in rural southwest China.

## 1. Introduction

Type 2 diabetes mellitus causes significant morbidity and mortality worldwide, with the prevalence of diabetes among adults aged ≥ 20 years estimated to an increase from 415 million in 2015 to 642 million by 2040 [1]. In addition to this substantial health burden, as a chronic metabolic disease, diabetes and its complications also impose a major economic burden upon societies both in direct healthcare costs and in indirect costs related to productivity loss. Diabetes-related social costs will continue to rise due to both the rapid rise in diabetes prevalence and ageing of the population [2, 3].

In China, type 2 diabetes mellitus is not only highly prevalent; its prevalence is increasing steadily, with the number of people with diabetes projected to increase from 20.8 million in 2000 to 42.3 million in 2030 [3]. Further, a recent study indicated that direct costs of diabetes in China have risen over time [4]. Clearly, interventions need to be made to prevent and control diabetes.

Previous attempts to estimate the economic impact of diabetes in China have focused solely on the direct healthcare costs of treating diabetes, overlooking indirect costs related to productivity loss as well as the financial impact of diabetes on the families of those afflicted with the disease. Moreover, the exact nature of the increase in economic burden of diabetes in China over time remains poorly understood. As diabetes prevalence rapidly rises and poverty due to illness becomes a growing social problem in rural regions, it is critical to understand the changing dynamics of diabetes and its related costs in China, especially in rural areas [5, 6].

The aim of the present study was to use a prevalence-based cost-of-illness methodology from a societal perspective (including analysis of both direct and indirect costs) to uncover trends in the change in economic burden of type 2 diabetes over time and estimate its financial impact on households in rural Yunnan Province, China, from 2009 to 2016.

## 2. Methods

### 2.1. Data Sources and Study Population

Yunnan Province is located in southwest China and as of 2014 had a population of 47.14 million. While Yunnan is one of the least developed provinces in China, it is one of the most diverse: Yunnan is home to 25 of China's ethnic minority groups. The study population included residents who responded to Yunnan Provincial Community Health Survey (YCHS) cycles 1.1 (2009) and 2.1 (2016). Data from two cross-sectional YCHS cycles with independent samples were combined, so temporal trends could be examined across two time periods: 2008–2009 and 2015–2016.

The research sample for the present study was selected in 2009 (*n* = 6350) using a multistage stratified random sampling method. The sampling method has been described in detail previously [7]. In 2009, a four-stage stratified random sampling method was used to select the study sample. In the first stage, the 129 counties in Yunnan were divided into two categories (economically advantaged and economically disadvantaged) according to wealth distribution (GDP per capita). From each of these two categories, one county was randomly selected, for a total of two counties. In the second stage, each selected county was further classified into three categories according to per capita GDP: low, medium, and high. From each of these three categories, one township was then randomly selected, for a total of six townships. In the third stage, three villages were chosen by probability proportional to size (PPS) from each of the 6 townships. In the final stage, in each selected village, we first obtained a name list of individuals aged ≥ 35 years and have lived in a village more than five years from the village committee, then simple random sampling was done with random number tables to select the individuals from this village. In 2016, the survey employed consistent four-stage stratified random sampling to select study subjects from the two rural areas (*n* = 6359).

### 2.2. Data Collection and Measurement

The two surveys used similar questionnaires to collect data from the participants. Both in 2009 and in 2016, trained interviewers used a pretested structured questionnaire to individually interview each consenting participant in person. Both household and individual level data were collected. Information on household income, household food expenditure, individual demographic characteristics, and diagnosis, treatment, and self-reported family history of diabetes was obtained. Information on inpatient hospitalization expenditures and outpatient expenditures was recorded from medical records of healthcare institutions, while information on direct nonmedical costs, self-medication costs, and work absence due to diabetes was collected by patient's self-report for the previous 12-month period for subjects who reported a previous diagnosis and/or treatment of diabetes by a medical doctor and who were using one or more antidiabetic medication at the time of the present study. The questionnaire includes a detailed list of the diabetes-specific direct nonmedical costs (including expenditures for hired caregivers and nutritional supplements, costs of transportation, and costs for accommodations on hospital visits), self-medication costs, and work absence due to diabetes. For example, we asked the question in the questionnaire: “How much money did you and your family spend on travel expenses and accommodations while you were in the hospital?”.

Local healthcare workers at community clinics measured each participant's fasting blood sugar (FBG). These blood samples were obtained from participants in the morning after completion of an overnight fast of a minimum of eight hours. Those participants reporting to be nonfasting were asked to return on a future date for FBG testing.

### 2.3. Ethical Approval

The Ethics Committee of Kunming Medical University approved this study prior to the commencement of research.

### 2.4. Cost Calculation

The present study calculated the cost of diabetes as the sum of direct and indirect costs. The estimated 2009 costs of diabetes were converted into 2016 constant Chinese Yuan (RMB) by multiplying by 1.05 based on the Consumer Price Index (CPI) [8] for 2009 and 2016 in China. The 2016 exchange rate of $1 = 6.14 RMB was used to calculate costs in US dollars.

### 2.5. Calculation of Direct Costs

Direct costs were subcategorized into direct medical costs and direct nonmedical costs. Direct medical costs included those for medications, outpatient visits, and hospital inpatient diabetes treatment. Direct nonmedical costs included expenditures for hired caregivers and nutritional supplements, as well as costs of transportation and accommodation when visiting healthcare providers by both the patient and his/her accompanying relatives. The detailed method of calculation of direct costs used has been previously described [8].

### 2.6. Calculation of Indirect Costs

Indirect costs referred to the costs of productivity losses due to diabetes-related morbidity (morbidity cost). A standard human capital approach was used to calculate morbidity cost [9], multiplying total number of days absent from work as a result of diabetes for both the patient and the relevant relatives by the 2009 and 2016 average daily gross earning per person. Income data were obtained from the 2009 and 2016 Yunnan Statistical Yearbook [10, 11], in which the reported annual per capita net income of rural households was 3102 RMB (US$505) in 2009 and 9892 RMB (US$1,611) in 2016. The average reimbursement rate for inpatient services made by the Chinese new rural cooperative health insurance scheme (NCMS) was 50% in 2009 and 60% in 2016.

An estimation of total community cost was determined by multiplying the overall cost calculated for the sample group by the ratio of the total community population divided by the sample population.

### 2.7. Definitions

Diabetes mellitus was defined as either a participant report of antidiabetic medication use within the prior two-week period or a lab result obtained of FGB ≥ 7.0 mmol/l (126 mg/dl). Treatment of diabetes was defined as a participant reporting the use of an antidiabetic medication (insulin and/or an oral hypoglycemic) within one month before the survey.

Households with health payments equaling or exceeding 40% of their nonfood expenditures or capacity to pay for services in 12 months before the interview were defined as incurring catastrophic health payment [12].

The 2016 Chinese national poverty line, 2300 Yuan (US$375) per capita per year, was used to assess the impoverishing effect of health payments. When per capita expenditure within the household decreased to below that of the established poverty line after health payments, households were defined as impoverished due to medical expenses [13].

### 2.8. Statistical Analysis

R3.2.0 software was used for all data analyses [14]. Categorical variables were presented in terms of frequency and percentages. Comparison of categorical variables between survey years was computed with a chi-square statistic. A *t*-test was used to estimate continuous variables between survey years. The overall treatment rates and prevalence of diabetes were adjusted for age and sex by direct standardization to the 2010 Chinese adult population aged ≥ 35 years. Two-tailed *P* values were used for statistical significance determination.

## 3. Results

A total of 3750 households involving 6600 individuals in 2009 and 3920 households involving 6600 individuals in 2016 aged ≥ 35 years were invited to participate in this study. Of these, 6350 in 2009 and 6359 in 2016 consented, for an overall response rate of 96.2% and 96.3%, respectively.

Table 1 shows the demographic characteristics of the study population by survey year. The participant population in 2009 consisted of 3067 males and 3283 females and in 2016 were 3143 males and 3216 females. There were no significant differences between the two survey years in mean age, proportion of males vs. females, or proportion of participants with low annual household income (*P* > 0.05). However, the adult illiteracy rate for women decreased from 59.0% in 2009 to 38.7% in 2016 (*P* < 0.01). In both 2009 and 2016, females had a lower annual household income rate than males and a higher illiteracy rate than males (*P* < 0.05).

The overall prevalence and treatment rates of diabetes increased from 7.7% and 20.0% to 9.5% and 32.1%, respectively (*P* < 0.01), over the seven-year interval from 2009 to 2016. Moreover, the increasing rates were observed in both males and females (*P* < 0.01). However, both in 2009 and in 2016, the prevalence of diabetes was higher in females than males (*P* < 0.05).

Table 2 presents the cost components of diabetes by sex and survey year. Total cost of illness of diabetes was estimated at $88 million in 2009 and $133.6 million in 2016. Males had higher overall direct and indirect costs of diabetes than females (*P* < 0.05) both in 2009 and in 2016. The largest component of the economic cost of diabetes was direct costs. Specifically, hospital costs were the main driver of direct medical expenditures, accounting for 66.2% of the total direct costs in 2009 and 75.9% in 2016. From 2009 to 2016, the total economic cost of diabetes increased 1.52-fold, with the largest increases observed in hospital costs (1.77-fold increase). In contrast, unit medication costs fell by 18.6%.

Table 3 shows that the total number of households including a member with diabetes was 262 in 2009 and 313 in 2016, respectively. Of these, 63 and 74 households faced financial catastrophe due to diabetes, and 47 and 55 households were impoverished by diabetes-related health spending in 2009 and 2016, respectively. Accordingly, the incidence of household catastrophic health payments due to diabetes was calculated at 24.0% in 2009 and 23.6% in 2016 among the surveyed communities, and the incidence of medical impoverishment due to diabetes was 17.9% and 17.6% in 2009 and 2016, respectively. Incidence of household catastrophic health payments due to diabetes and incidence of medical impoverishment due to diabetes did not differ between the two survey years (*P* > 0.05).

## 4. Discussion

The findings indicate that diabetes is a significant and growing public health challenge in rural southwest China as both its morbidity and economic burden increased considerably over the seven-year study period.

In this study, the overall prevalence rate of diabetes was greater than prevalence rates observed in other parts of rural China [15, 16], whereas the overall treatment rate was well below that found in previous studies [16]. The present study also uncovered sex differences in the prevalence of diabetes in the study region as women had a higher prevalence than men. This finding accords with prior Chinese studies [16, 17] as well as studies in other Asian countries, including Thailand and India [18, 19]. Moreover, the results indicate that the prevalence of diabetes has increased substantially both in men and in women in rural Yunnan Province, consistent with trends observed in a previous Chinese study [16]. The findings also suggest that diabetes is a major public health problem in terms of morbidity in rural southwest China. This presents a challenge for local governments to take measures to improve the early detection and prevention of diabetes in order to forestall an emerging epidemic.

Further, the present study revealed gender differences in the costs of diabetes in rural Yunnan Province. Namely, both in 2009 and in 2016, males with diabetes were more likely to have both higher direct and indirect costs of diabetes than females. This likely results from the fact that females with diabetes were less able to afford adherence to recommended medication than males in the surveyed communities. In addition, as males are the primary labor force in rural China, diabetes had a greater impact on the expense of productivity loss due to morbidity for males than for females. The findings suggest that females are a vulnerable population in rural China and should be given critical consideration when determining appropriation of financial resources for diabetes treatment programs.

Among the study population, direct medical costs comprised the largest component of overall costs related to diabetes (over 87%) both in 2009 and in 2016, while inpatient hospitalization was the main cost driver of direct expenditures (over 62%). This finding differs from other studies conducted in Singapore and Thailand [20, 21], in which indirect costs accounted for the largest proportion of total costs of diabetes. This is possibly because cost of productivity loss due to premature mortality was not included in the indirect costs in our study. However, due to the wide variation in the methodology of cost estimations and the cost components chosen to be included and analyzed in various studies, it is difficult to directly compare our results with other similar studies.

The present study indicated that the direct, indirect, and total economic costs of diabetes increased over time. Specifically, the largest increases were observed in hospital costs of diabetes, while a decreasing trend was found for medication costs. The unit hospital cost per person with diabetes both in 2009 and in 2016 (US$709.50 and US$1016.20) was much higher than average annual household income (US$539 and US$836), indicating hospital care services impose a heavy financial burden on rural households in China and families are vulnerable to becoming impoverished due to a family member's diabetes-related hospital admittance. Moreover, the overall incidence rates of household catastrophic health payment and household impoverishment due to diabetes in 2009 and 2016 in our study were both significantly higher than the Chinese national average rates (13.0% and 7.5%) [22] as well as rural rates from a previous Chinese study (14.4% and 9.2%) [22]. Encouragingly, however, the present study found that although costs of diabetes increased significantly over the seven-year study period, incidence of household catastrophic health payment and impoverishment due to diabetes did not differ between the two survey years. This may be attributable to the progress made by the new rural cooperative health insurance scheme (NCMS) [23] in protecting citizens from financial catastrophe and impoverishment in rural southwestern China. Yet, the findings suggest that higher reimbursement for inpatient services is urgently needed for the NCMS policy to prevent rural households from medical impoverishment.

The present study is limited in several respects. First, the diagnosis of diabetes among study participants was solely based on FBG levels; haemoglobin A1C was not measured, which could have caused an underestimation of the prevalence of diabetes. Second, the recorded figures for direct costs related to diabetes were based on patient recall and may therefore be subject to bias. Third, only the costs of diabetes itself were considered; the present study did not include the costs of diabetes-related complications. It is probable that by excluding such complication-related costs, the costs of diabetes have been underestimated. Fourth, the cost estimates used in the present study did not include premature death due to diabetes, so the study may have further underestimated the true economic burden of diabetes.

In conclusion, both the prevalence and economic burden of diabetes and catastrophic expenditure due to diabetes are immense in rural southwest China. Diabetes has become a major public health problem with both morbidity and economic burden markedly increasing from 2009 to 2016. The findings underscore an urgent need for greater financial investment in the prevention of diabetes as well as improvement of access to affordable medication in rural southwest China.

## Figures and Tables

**Table 1:** General characteristics of the study population by survey year.

Characteristics	Survey year					
	2009			2016		
	Male (*n* = 3067)	Female (*n* = 3283)	All (*n* = 6350)	Male (*n* = 3143)	Female (*n* = 3216)	All (*n* = 6359)
Age (%)						
35-44 years	712 (23.2)	761 (23.2)	1473 (23.2)	737 (23.4)	714 (22.2)	1451 (22.8)
45-59 years	1176 (38.3)	1262 (38.4)	2438 (38.4)	1141 (36.3)	1285 (40.0)	2426 (38.2)
≥60 years	1179 (38.4)	1260 (38.4)	2439 (38.4)	1265 (40.2)	1217 (37.8)	2482 (39.0)
Level of annual household income (%)						
Low	1230 (40.1)	1768 (53.9)^∗^	2998 (47.2)	1264 (40.2)	1614 (50.2)^∗^	2878 (45.3)
High	1837 (59.9)	1515 (46.1)	3352 (52.8)	1879 (59.8)	1602 (49.8)	3481 (54.7)
Education level (%)						
Illiterate	740 (24.1)	1671 (59.0)^∗∗^	2411 (38.0)	856 (27.2)	1244 (38.7)^∗∗^	2100 (33.0)
Primary (grade 1-6) or higher	2327 (75.9)	1612 (49.1)	3939 (62.0)	2287 (72.8)	1972 (61.3)	4259 (67.0)
Mean fasting plasma glucose (mmol/l)	5.2 ± 1.5	5.4 ± 1.7	5.3 ± 1.8	5.7 ± 1.6	5.8 ± 1.7	5.8 ± 1.7
Age-standardized prevalence of diabetes (%)	6.3	9.1	7.7	8.4^∗^	11.2^∗∗^	9.5^∗∗^
Age-standardized treatment rate of diabetes (%)	16.1	22.7	20.0	29.9^∗∗^	34.3^∗∗^	32.1^∗∗^

^∗^ *P* < 0.05, ^∗∗^*P* < 0.01.

**Table 2:** Cost of illness of diabetes (in US$) by survey year in rural Yunnan Province, China.

Cost components	2009					2016				
	Unit cost			Total	% of total cost of illness	Unit cost			Total	% of total cost of illness
	Male	Female	All			Male	Female	All		
Direct medical costs	1168.1	995.3	998.0	77,193,729.0	87.8	1326.8	1237.5	1273.8^∗∗^	121,436,449.2	90.9
Outpatient visits	62.3	38.5	48.0	3,709,008.0	4.3	65.2	34.5	45.5	4,337,697.0	3.2
Hospitalizations	866.6	713.4	709.5	54,823,774.5	62.3	1006.8	1029.0	1016.2^∗∗^	96,878,410.8	72.5
Medications	239.2	253.4	251.5	18,660,946.5	21.2	254.8	174.0	212.1^∗^	20,220,341.4	15.1
Direct nonmedical costs	111.6	59.0	73.0	5,640,783.0	6.3	71.1	34.5	65.5	6,244,377.0	4.7
Direct costs	1279.7^∗^	1054.3	1072.0	82,834,512.0	96.3	1397.9^∗^	1272.0	1339.3	127,680,826.2	95.6
Indirect costs	61.2^∗^	32.1	55.1	5,168,111.0	5.9	64.8^∗^	51.3	62.0	5,910,708.0	4.4
Total cost of illness	1335.3	1083.5	1113.0	88,002,623.0	100.0	1462.7^∗^	1323.3	1401.3	133,591,534.2	100.0

^∗^ *P* < 0.05, ^∗∗^*P* < 0.01.

**Table 3:** Incidence of financial catastrophe and household impoverishment due to diabetes by survey year in rural Yunnan Province, China.

Variable	2009		2016	
	*n*/*N*	% (95% CI)	*n*/*N*	% (95% CI)
Catastrophic health payment	63/262	24.0 (18.9, 29.2)	74/313	23.6 (19.3, 28.7)
Household impoverishment	47/262	17.9 (13.8, 23.0)	55/313	17.6 (13.8, 22.2)

## Data Availability

The datasets used and/or analyzed during the current study are available from the corresponding author upon request.
